# Tannic Acid-Lung Fluid Assemblies Promote Interaction and Delivery of Drugs to Lung Cancer Cells

**DOI:** 10.3390/pharmaceutics10030111

**Published:** 2018-08-01

**Authors:** Elham Hatami, Prashanth K. B. Nagesh, Pallabita Chowdhury, Subhash C. Chauhan, Meena Jaggi, Amali E. Samarasinghe, Murali M. Yallapu

**Affiliations:** 1 Department of Pharmaceutical Sciences and Center for Cancer Research, University of Tennessee Health Science Center, Memphis, TN 38103, USA; ehatami@uthsc.edu (E.H.); pbhusett@uthsc.edu (P.K.B.N.); pchowdhu@uthsc.edu (P.C.); schauha1@uthsc.edu (S.C.C.); mjaggi@uthsc.edu (M.J.); 2 Department of Paediatrics, University of Tennessee Health Science Center, Memphis, TN 38103, USA; asamaras@uthsc.edu

**Keywords:** tannic acid, lung fluid, protein corona, lung cancer, cancer therapeutics

## Abstract

Lung cancer (LC) is one of the leading causes of death in both men and women in the United States. Tannic acid (TA), a water-soluble polyphenol, exhibits a wide range of biological activities. TA has received much attention as a promising compound in the biomaterial and drug delivery fields. Lung fluid (LF) is a major barrier for distribution of drugs to the lungs. Therefore, the purpose of this study was to examine TA interaction with LF for effective delivery of anti-cancer drug molecules via pulmonary delivery. The extent of adsorption of LF proteins by TA was revealed by fluorescence quenching in fluorescence spectroscopy. The presence of LF in TA-LF complexes was noticed by the presence of protein peaks at 1653 cm^−1^. Both protein dot and SDS-PAGE analysis confirmed LF protein complexation at all TA concentrations employed. A stable particle TA-LF complex formation was observed through transmission electron microscopy (TEM) analysis. The complexation pattern measured by dynamic light scattering (DLS) indicated that it varies depending on the pH of the solutions. The degree of LF presence in TA-LF complexes signifies its interactive behavior in LC cell lines. Such superior interaction offered an enhanced anti-cancer activity of drugs encapsulated in TA-LF complex nanoformulations. Our results indicate that TA binds to LF and forms self-assemblies, which profoundly enhance interaction with LC cells. This study demonstrated that TA is a novel carrier for pharmaceutical drugs such as gemcitabine, carboplatin, and irinotecan.

## 1. Introduction

Lung and bronchial cancer is the most lethal cancer in the United States with 234,030 estimated new diagnoses and 154,050 deaths in 2018 [1,2]. Chemotherapy is the most common treatment regimen for lung cancer (LC). Most common therapeutic drugs for LC treatments are carboplatin, cisplatin, gemcitabine, paclitaxel, and irinotecan. The efficacy of treatment mostly depends on the mechanism by which the drug is delivered and the optimum concentration of the drug available in the tumor cells. Conventional chemotherapy has poor distribution and results in only a limited amount of therapeutic agent(s) reaching tumor cells, due to lung fluid (LF), a biological barrier that restricts drug penetration to the lungs. There is a significant amount of research dedicated to the study of drug penetration to lung tumors. Often, the LF corona is a major determining factor on how these therapeutic agents/complexes behave in in vivo applications. This is a growing field of research aimed at understanding drug carrier stability and fate when the carriers encounter proteins in biofluids. These studies direct the research towards reducing uncertainties involved in the prediction of the behavior of therapeutic pharmaceutical nanoformulations in in situ and in vivo, and their application in clinical trials.

Tannic acid (TA) is a polyphenolic compound and an important form of water-soluble tannin. It is widely found and commercially extracted from plants such as tara (*Caesalpinia spinosa*), gallnuts (*oak gall*), and Sicilian sumac (*Rhus coriaria*). TA is a mixture of galloyl esters and their derivatives, and it is represented with the chemical formula C_76_H_52_O_46_. TA is a useful diagnostic tool as a dye that is used to stain plasma membranes, particularly binding most prominently to the margins of tumors [3]. This suggests its binding and targeting efficiency to cancer cells. TA alone was able to protect against benzo(a)pyrene-induced LC in rats [4]. Considering these outcomes as patentable innovation, there was an invention allowed to develop tannin complexes or mixtures with TA and pharmaceutical compositions [5]. There is substantial evidence that TA is an important biocompatible and biodegradable compound commonly used for biomedical and clinical applications [6,7,8]. The abundance of hydroxyl functional groups in TA can offer increased solubilization, complexation, entrapment, and encapsulation of therapeutic cargo for slow and sustained release applications [9,10,11]. TA has been applied as a carrier for low molecular weight drugs (paclitaxel, docetaxel, amphotericin B, curcumin, and rapamycin) [9,10,11,12] and biomacromolecules (vaccines, peptides, antibodies, and DNA) [13,14,15,16,17]. Additionally, TA has been known to crystalize various active pharmaceutical ingredients [18]. Pre-clinical evidence has shown that TA has significant anti-cancer and chemo-preventive profiles [19]. However, its application in LC has never been studied. All these events suggest that TA-based delivery would enhance delivery of therapeutics and would therefore have relevance in LC.

Drug carrier interaction with serum proteins, which results in a carrier-protein corona, is a predictor for in vivo stability and applicability [20,21,22,23]. This behavior is common in the case of intravenous and oral administration of nanotherapeutics. TA in wine is passively absorbed by serum proteins and has not shown side effects. Our ultimate objective is to develop TA as a therapeutic carrier to improve bioavailability and targetability of active pharmaceuticals applied to treatments for lung cancer. The intent of this formulation is to apply for nasal or pulmonary delivery. Therefore, in this study, we aimed to investigate the interaction of TA with LF. The interaction studies were investigated by fluorescence and Fourier Transform Infrared (FTIR), protein dot, SDS-PAGE, DLS, and transmission electron microscopy (TEM). The LF protein corona influence on cellular internalization was examined by fluorescence spectroscopy and flow cytometry. Improved pharmaceutical efficacy through TA-based assembly was confirmed by cell proliferation assay.

## 2. Materials and Methods

### 2.1. Materials

All reagents and chemicals stated below were purchased from Fisher Scientific (Pittsburgh, PA, USA) or Sigma-Aldrich Co. (St. Louis, MO, USA), unless further information is presented. Bronchoalveolar lavage fluid of mice (simply termed as LF) was provided by Dr. Amali Samarasinghe (UTHSC, Memphis, TN, USA) collected under Institutional Animal Care and Use Committee (IACUC) approved protocol. For this, six-week-old female C57BL/6 mice were purchased from Jackson Laboratories (Bar Harbor, ME, USA), and housed in microisolator cages with alpha-dri bedding and free access to mouse chow and autoclaved water in a climate-controlled room for acclimatization. At 12–15 weeks of age, mice were euthanized by CO_2_ asphyxiation and the trachea surgically cannulated. The diaphragm was incised, and bronchoalveolar lavage performed with two 1 mL aliquots of sterile phosphate-buffered saline (PBS). Cells were separated by centrifugation at 600× *g* for 10 min at 4 °C, and the fluid was stored at −80°C until use. 

### 2.2. TA-LF Complexation

TA and LF interaction leading to self-assembly/complexation was investigated by analysis of LF protein corona formation on TA molecules. For this, 30 μg of mouse LF was incubated with various concentrations of TA for 1 h in 2 mL Eppendorf tubes. According to our previous reports, protein corona formation takes place after 30 min [22,23]. The tubes were centrifuged in Sorvall ST 8 Centrifuge at 5000 rpm (Thermo Fisher Scientific, Suzhou, China) for 2 min to separate any large clumps. This fine TA-LF complex suspension was used for all studies. Both TA alone and LF alone were used as controls, separately.

### 2.3. Fluorescence Spectroscopy

The binding ability of LF protein tryptophan residues with TA was evaluated by measuring fluorescence quenching using fluorescence spectrometry with a SpectraMax Plus plate reader (Molecular Devices, Sunnyvale, CA, USA). The fluorescence emission spectra were recorded between 250 and 400 nm (slit at 5 nm) at an excitation wavelength 295 nm (slit at 2 nm). Thirty micrograms of LF in 1 mL 1× PBS buffer in a quartz cuvette was used for the fluorescence quenching experiment. After running this solution as a control sample, titration was performed with 5, 10, 15, 20, 25, 30, and 40 µg of TA by successive addition of 1 μL of TA (5 mg/mL). This assay was performed in triplicate.

### 2.4. Fourier Transform Infrared Spectroscopy

Fourier transform infrared (FTIR) spectroscopy was employed to confirm the presence of LF proteins in TA-LF complexes. Ten microliters of TA-LF complex solutions were placed on a Diamond/ZnSe Attenuated Total Reflection crystal plate and air dried, then spectra were acquired using a PerkinElmer Spectrum 100 FTIR spectrometer (Waltham, MA, USA). The spectra were recorded for these samples and TA/LF samples (controls for peaks comparison) from 4000 to 650 cm^−1^ with a resolution of 4 cm^−1^. FTIR spectra were presented as an average of 32 scans for all samples. 

### 2.5. Protein Density and SDS-PAGE Gel of TA-LF Complexes

Following TA and LF complexation, the TA-LF complex solution was centrifuged at 1000 rpm (Sorvall ST 8 Centrifuge) to obtain stable TA-LF self-assemblies. Ten μL of these complex solutions, were transferred onto a nitrocellulose membrane (Bio Rad, Hercules, CA, USA) and the samples were allowed to diffuse protein into the membrane and dry at room temperature. Following a rinse with DI water, the membrane was incubated in 5 mL of 0.25% Coomassie Blue R-250 dye (#20278, Thermo Fisher Scientific) (50:10:40 v% methanol:glacial acetic acid:DI water). The nitrocellulose membrane in Coomassie Blue solution was heated in a 1200 W microwave for 30 s After cooling to room temperature, the dye solution was drained, and the membrane washed twice with DI water, then de-stained in 50:10:40 v% methanol:glacial acetic acid:DI water solution without dye until it displayed a clear background. After a final rinse with DI water, the blue spot protein-stained membrane was stored in DI water at room temperature and image captured using a camera. This assay was performed in triplicate. Similarly, these TA-LF complexes were used for generating SDS-PAGE gels according to our previous protocol [22]. 

### 2.6. Particle Size and Zeta Potential

The intensity-weighted average particle size (nm), particle distribution, and polydispersity index (PDI) of samples were measured with a dynamic light scattering method using a Zetasizer instrument (Nano ZS, Malvern Instruments Ltd., Worcestershire, UK). To acquire these measurements, 20 µL of each sample solution was added to 1000 µL of filtered Milli-Q water and probe sonicated (VirSonic Ultrasonic Cell Disrupter 100, VirTis, Woburn, MA, USA) for 30 s. Particle size measurements were performed at 25 °C for 3 min. An average of three readings for each solution was calculated and recorded. Zeta potential (ζ, mV) of the particles was quantified with laser Doppler velocimetry using the same instrument. Samples were diluted with 1× PBS and measured for 30 runs each. Data were recorded as an average of three readings. These measurements were also acquired in solutions of different pH.

### 2.7. Particle Morphology

The particle size and morphology of TA-LF complexes were examined by using TEM (JEOL 200EX, JEOL Ltd., Tokyo, Japan). For this investigation, after probe sonication of the TA-LF complex solution for 30 s, 20 μL of solution was dropped carefully on the shiny side of the 200-mesh standard TEM grid (#FCF200-CU-SB, Electron Microscopy Sciences, Hatfield, PA, USA). UranyLess EM Stain solution (22409, Electron Microscopy Sciences) was used as a positive contrast agent to achieve a stronger contrast between the background and the nanoparticles. Morphology examination of TA-LF particles was performed on air-dried grids at 80 kV accelerating voltage under an advanced microscopy digital camera imaging system (Advanced Microscopy Techniques, Corp., Woburn, MA, USA). 

### 2.8. Cell Culture, Growth, and Condition

The human LC cell lines A549 (carcinoma cell line derived from 58-year-old male Caucasian, #ATCC^®^ CCL-185™) and H1299 (carcinoma non-small cell LC cell line derived at the metastatic lymph node from 43-year-old adult, #ATCC^®^ CRL-5803™), were purchased from American type culture collection (Manassas, VA, USA). These cell lines were expanded, grown, and used at low passages for experiments. The expanded cell line aliquots (less than 5–6 passages) were stored frozen in liquid nitrogen for future usage. The cancer cell lines cultured under sterile condition in Dulbecco’s Modified Eagle’s medium (DMEM for A549) and Roswell Park Memorial Institute medium (RPMI for H1299) supplemented with 4.5 g/L of glucose, 10 nM of nonessential amino acids (# 11140076, Gibco, Thermo Fisher Scientific, Grand Island, NY, USA), 100 mM of sodium pyruvate (#11360070, Gibco), 1× antibiotic/antimycotic (#15240062, Gibco), and 10% heat-inactivated FBS (#10438026 Thermo Fisher). Cells were maintained in 100 mm tissue culture dishes (#83.3902, Sarstedt, Inc., Newton, NC, USA) as 2D monolayers in a humidified incubator (5% CO_2_ and 95% air condition) at 37 °C (Thermo Fisher Scientific, Waltham, MA, USA). 

### 2.9. Cellular Uptake

A semi-quantitative cellular uptake assay of TA-LF complexes was examined by flow cytometry and fluorescence microscopy methods. To allow cellular uptake quantification, TA-LF assemblies were labeled with coumarin-6 using our previously reported method [24,25]. The extent of dye internalized was used to track uptake of TA-LF self-assemblies into cells. 100 μg of coumarin-6 dye was loaded in 1 mg of TA-LF self-assembled. In this study, 2 mL of media containing 5 × 10^5^ cancer cells per well were seeded in 6-well plates (#83.3920.005, Sarstedt, Inc.) and cells were allowed to attach to plates overnight, then were dosed with 5 μg coumarin-6 containing TA-LF particles with different densities of LF for 30 min to 6 h. After the incubation time, cells were washed twice with 1× PBS and replaced with fresh phenol red-free media to each plate. The uptake of TA-LF particles was imaged using an EVOS^®^ FL Imaging System (AMF4300, Life Technologies, Carlsbad, CA, USA) for visual comparison. For quantitative measurements, the cells were trypsinized, then centrifuged at 1000 rpm for 5 min and resuspended in 2 mL phenol red-free media. About 10,000 cells from these cell suspensions were used to acquire fluorescence levels using an Accuri C6 flow cytometer (Accuri Cytometer, Inc., Ann Arbor, MI, USA). The measurements were performed in triplicate using FL1 channel (488 excitation, Blue lase, 530 ± 15 nm, FITC/GFP).

### 2.10. MTS Assay

The drugs (gemcitabine, carboplatin, irinotecan) encapsulated in TA-LF formulations were prepared following our established protocol [24]. Their anti-cancer efficacies were evaluated using a colorimetric cell titer 96 aqueous one solution cell proliferation assay (MTS assay, Promega, Madison, WI, USA). In this study, LC cell lines (A549 and H1299) were plated at 5 × 10^3^ cells/well in 96-well flat bottom tissue culture plates (#83.3924.005, Sarstedt, Inc.) and incubated at 37 °C in a 5% CO_2_/95% air atmosphere overnight for attaching to plate. Cells were treated with free drugs (gemcitabine, carboplatin, and irinotecan) and equivalent drug-containing TA-LF formulations. Respective cell lines with no treatment or TA alone were used as controls. After 48 h treatment, 20 μL of MTS reagent solution was added to each well and incubated for 2 h at 37 °C. Absorbance was measured at 490 nm by a Cytation 5 imaging microplate reader (BioTek, Winooski, VT, USA). Cell proliferation was normalized to that of cells cultured in medium with no treatment. 

### 2.11. Statistical Analysis

All measurements and results generated in this study were presented as mean ± standard error of mean (SEM). The statistical analysis and significance of data were assessed using Student’s *t*-test; significance was set at *p* < 0.05 with GraphPad 5.03 Prism program (La Jolla, CA, USA).

## 3. Results and Discussion

Efficient delivery of therapeutics remains the most desirable outcome for treating LC. However, successful delivery is always compromised by poor penetration, quick elimination, and poor bioavailability. This phenomenon is partly due to the abundance of LF, which determines the fate of the drug. Greater interaction with LF may lead to cross-linking with proteins, and thus a huge foreign particle can develop and be eliminated by the system. Therapeutic formulations with limited interaction with LF proteins and unique complexation/self-assembly formation may improve circulation and deep penetration into lungs. Considering this hypothesis, TA is proposed as a carrier for LC therapeutics, and thus this study aims to examine its specific interaction with LF. The outcome of this study would provide a new therapeutic approach for LC. Further, this study will also explore the possible therapeutic role of drugs encapsulated within TA-LF self-assemblies.

### 3.1. Fluorescence Binding

**Figure 1:**
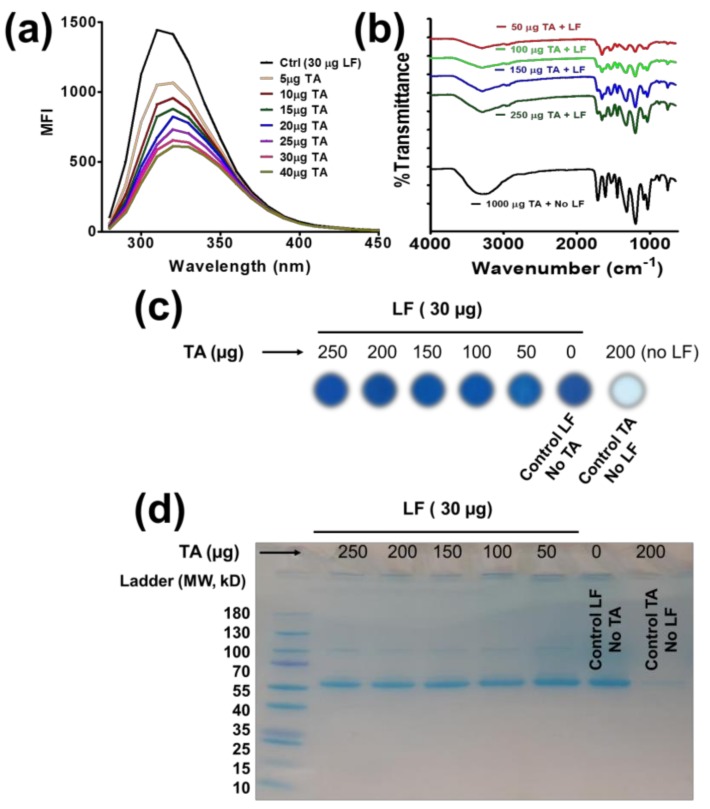
Spectral and biological confirmation of TA-LF complex formation. (**a**) Instant binding and self-assembly/complex formation of LF and TA was measured by measuring fluorescence (FL) quenching. Representative FL quenching profiles of 30 µg/mL LF with increased TA solution (5–40 µg) at room temperature (λex = 295 nm). (**b**) FTIR spectra of TA and TA-LF complexes. Characteristic peaks at 1653 and 1525 cm^−1^ represent the presence of LF in TA-LF complexes. (**c**,**d**) Protein dot and SDS-PAGE confirms presence of LF proteins in TA-LF complexes by visual evidence. Coomassie Blue stains lung fluid protein in TA-LF assemblies but not free tannic acid. (**a**,**c**,**d**) Data acquired from 3 sets of samples for confirmation.

First, we tried to delineate the TA-LF complexation process using the fluorescence (FL) quenching of proteins present in LF that would interact with tannic acid. The fluorescence of proteins arises from tryptophan residues in proteins [26,27]. TA exhibited a dose-dependent fluorescence decay (Figure 1a). This is primarily due to inter/intra-molecular interactions of the hydroxyl groups of TA with the amine groups of proteins. Such interaction occurs from a complex formation between protein and TA, which is responsible for quenching of the intrinsic fluorescence of the two tryptophan residues (Trp-134 and Trp-212). 

These data suggest that TA interaction with LF proteins is caused by coating or protein corona formation. Further, at lower concentrations, TA exhibited a minor change in fluorescence decay, indicating little interactions, while significant quenching occurs when TA concentrations employed were 20 µg/mL and above. 

### 3.2. FTIR Spectral Analysis

FTIR spectral analysis allows for a quick and efficient confirmation of TA and LF proteins by their functional groups (Figure 1b). The spectra of TA and TA-LF complexes displayed characteristic peaks of TA at 3280 cm^−1^ due to phenolic O–H stretch of the hydroxyl groups, 1725 and 1697 cm^−1^ due to C–O stretch of the carboxyl groups, and 1082 cm^−1^ due to C–O–C vibrations. The additional absorption bands at 1016, 1066, and 1187 cm^−1^ are ascribed to the vibrations of substituted benzene rings in TA and TA-LF. The spectrum of LF alone shows characteristic peaks for the amide I band at 1655 cm^−1^ (mainly C–O stretch) and amide II band at 1525 cm^−1^ (C–N stretching and N–H bend) (Supplementary Information Figure S1). The other peaks at 3290 and 1075 cm^−1^ correspond to N–H and C–N stretch of the aliphatic amine moiety (Supplementary Information Figure S1). In the spectra of the TA-LF complexes, the specific peak at 1653 cm^−1^ belongs to the amide I band of LF, while other bands coincide with TA characteristic peaks (Figure 1b). The major amide I peak decreased relatively with increase of TA in TA-LF complexes.

### 3.3. LF Proteins in TA-LF Complexes

Control LF and the TA-LF complex samples show almost equivalent protein stain with Coomassie Blue on nitrocellulose membranes (Figure 1c). There is no strong signal observed for TA alone samples. To gain better insights of TA-LF complex formation, we separated supernatant and pellet of TA-LF complexes which are considered well-dispersed/perfect corona formation and loosely bound corona/aggregates of corona, respectively. Supernatant TA-LF complexes at TA:LF ratio(s), 30:250, 30:200, and 30:150 show more protein stain while 30:100 and 30:50 demonstrated almost nil. This indicates higher TA concentration helping to achieve a better protein corona (smaller TA-LF complex formation, Supplementary Information Figure S2). TA-LF complex pellets indicate protein stain at 30:150, 30:100, and 30:50 reflect due to poor or random corona formation (Supplementary Information Figure S2). Altogether, this indicates higher TA promotes smaller TA-LF complexes. The abundance of LF protein in TA-LF complex samples was observed in SDS-PAGE gel (Figure 1d) due to combination of smaller and bigger complex formation due to LF corona formation. A similar pattern of protein exists in these samples, which further confirmed the data shown in Figure 1c. 

### 3.4. Protein Corona Formation

**Figure 2:**
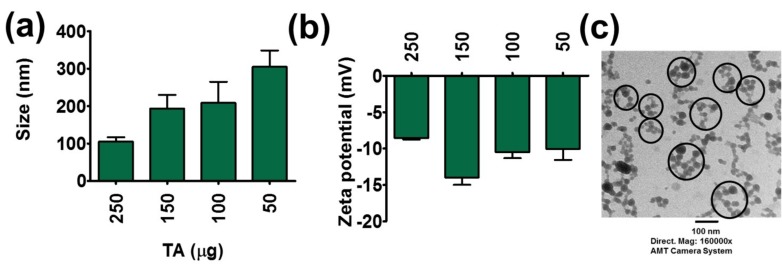
Physical characterization of TA-LF complexes. (**a**) Particle size and (**b**) zeta potential of TA-LF complexes. Measurements were performed using Zetasizer. Increasing LF inclusion in TA-LF alters protein corona on TA and the change in their particle size and zeta potentials. Data presented as mean ± SEM (*n* = 3). (**c**) Representative transmission electron microscopic image of TA-LF self-assemblies. Image was acquired using advanced microscopy digital camera system Print magnification 160,000×.

Protein corona formation is a biological phenomenon that occurs when nanoparticles or some other compounds are incubated with proteins. To investigate such phenomena, a DLS analysis method was employed. TA-LF complexes had particle size less than ~305 nm (Figure 2a). TA and LF alone showed a particle size of 340.8 ± 55.69 nm and 338.2 ± 5.64 nm, respectively (Supplementary Information Figure S3a). A higher amount of TA (250 μg) leads to lower particle size (105 ± 11.83 nm) (Figure 2a) due to hard protein corona on tannic acid [22]. Lower amounts of TA (50 μg) in TA-LF generated complexes with a particle size of 304.8 ± 43.64 nm. This suggests that TA concentration plays a major role in determining not only the globular protein corona formation, but also in determining particle sizes of complexes resulting from incubation with LF.

TA and LF alone exhibited low negative zeta potentials, −16.03 ± 0.17 mV and −7.68 ± 0.91 mV, respectively (Supplementary Information Figure S3b). When TA content increased in TA-LF assemblies, their zeta potential values are similar, as seen for LF zeta potential, i.e., −8.55 ± 0.20 mV for assembly resulting from 250 μg TA in a TA-LF assembly (Figure 2b). This is evidence that LF proteins are perfectly aligned on the TA core. When TA concentration is lower in TA-LF assemblies, the resulting zeta potential values lie between those for TA and LF (Figure 2b). This provides support for the hypothesis that these assemblies might be complexes of irregular formation, depending upon the amounts of TA employed. However, it is important to note that there is no positive zeta potential observed, which indicates that all TA-LF systems are stable, and complexation does not lead to huge aggregation or precipitation. Additionally, a negative zeta potential for nanosystem(s) or carrier(s) implies that these are safe for therapeutic applications, unlike a positive zeta potential for such materials, which induces systemic toxicity. 

TEM analysis was conducted to investigate the visual evidence for TA-LF complex formation and to observe the morphological status of each complex. TA-LF complexes exhibited a self-assembled spherical shape, about <30 nm with aggregations of <120 nm (Figure 2c). Such well-defined morphology of TA-LF nanoparticles can be an indication of effective penetration ability in lungs. Further, this indicates that TA is not inducing larger aggregates, which often cause elimination from circulation. 

**Figure 3:**
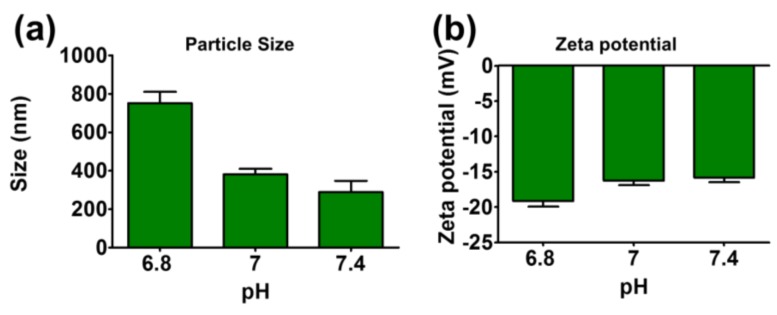
Influence of LF protein corona in TA-LF complexes with change in pH. (**a**) Particle size and (**b**) zeta potential of TA-LF were measured in different pH of 4-(2-hydroxyethyl)-1-piperazineethanesulfonic acid (HEPES) buffer solutions. Data presented as mean ± SEM (*n* = 3).

The particle sizes of TA-LF assemblies were tested at different pH solutions (6.8, 7, and 7.4) (Figure 3a). Interestingly, a significant increase in particle size was noticed at pH 6.8 (751.33 ± 32.26 nm) compared to all other pH measurements. This could be due to the loosening of proteins from the self-assemblies or protein corona on TA core. Such dissociation of self-assemblies may offer immediate release of therapeutics which is helpful in drug delivery. That means at tumor pH (around ~6.8) self-assemblies dissociate and release drugs for quick response or targeted delivery mechanism. It is also equally possible that TA is hydrolyzes more at this pH compared to other pH. However, zeta potentials were all negative for all pH tested (between −15.86 ± 0.76 mV and −19.13 ± 0.92 mV, see Figure 3b).

### 3.5. LF Protein Corona Promotes Interaction with LC Cells

It is essential for cancer therapeutic applications that drug carriers be able to internalize into the cancer cells, and the release the drug in the cytosol, to achieve a relevant beneficial therapeutic effect. 

**Figure 4:**
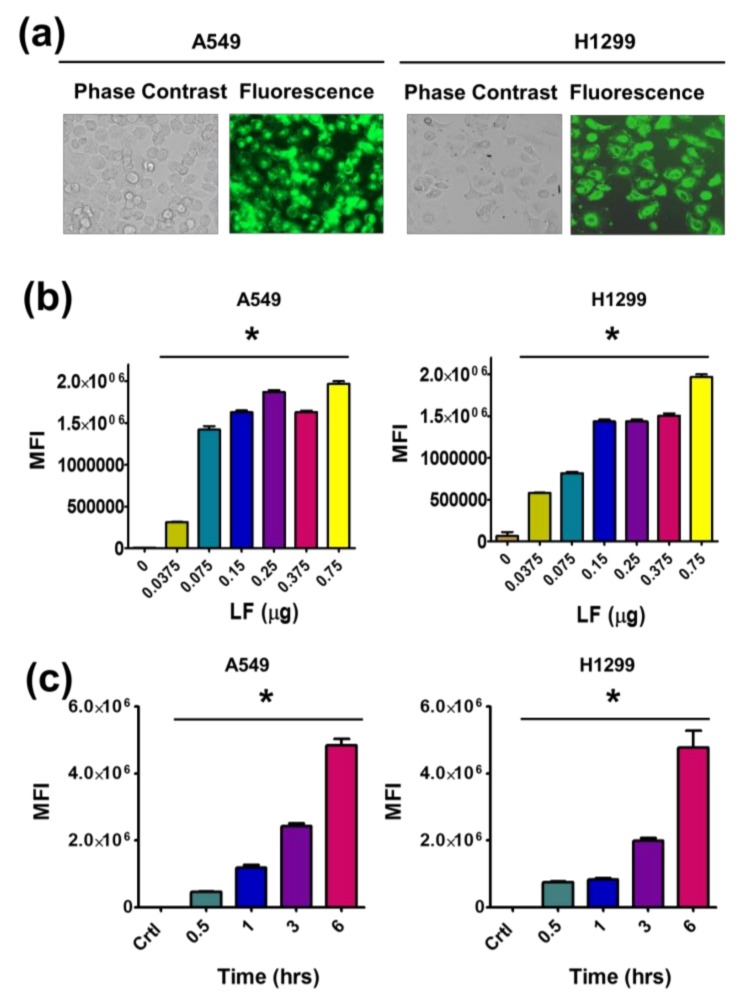
LF corona promotes cellular uptake of TA-LF complexes. (**a**) Cellular uptake of dye labeled TA-LF in A549 and H1299 cells. Cells were treated with coumarin-6 labeled TA-LF (5 μg coumarin-6 equivalent) for 3 h. Representative fluorescence image was presented. (**b**) LF corona on TA exhibited enhanced cellular uptake in A549 and H1299 cells. Coumarin-6 concentration in TA-LF was 5 μg/mL and incubation time was 3 h. (**c**) Time-dependent cellular uptake of coumarin-6 labeled TA-LF complexes. (**b**,**c**) FL levels of internalized coumarin-6 labeled TA-LF complexes were measured using an Accuri C6 Flow Cytometer in the FL1 channel. Data presented as mean ± SEM (*n* = 3). Uptake is significance compared to no LF or control cells (* *p*  <  0.05).

First, we investigated the uptake of TA-LF complexes by A549 and H1299 LC cells using fluorescence microscopy. Abundant intracellular distribution of TA-LF complexes is observed in cells through coumarin-6 labeling (Figure 4a). 

To achieve a global pattern of uptake of TA-LF complexes, flow cytometry was employed. For this, an average of 1 × 10^4^ cells was used to measure fluorescence intensities. An elevated intensity of green fluorescence was observed in both cell lines with increased amounts of LF protein in TA-LF self-assemblies used for the experiment (Figure 4b). Further, it was confirmed that this cellular interaction is time dependent (Figure 4c). With increased time of contact, the TA-LF complexes interact more with LC cells, due to the enhanced adherence of LF on the TA core.

### 3.6. TA-LF Improves Pharmaceutical Activity in LC Cells

**Figure 5:**
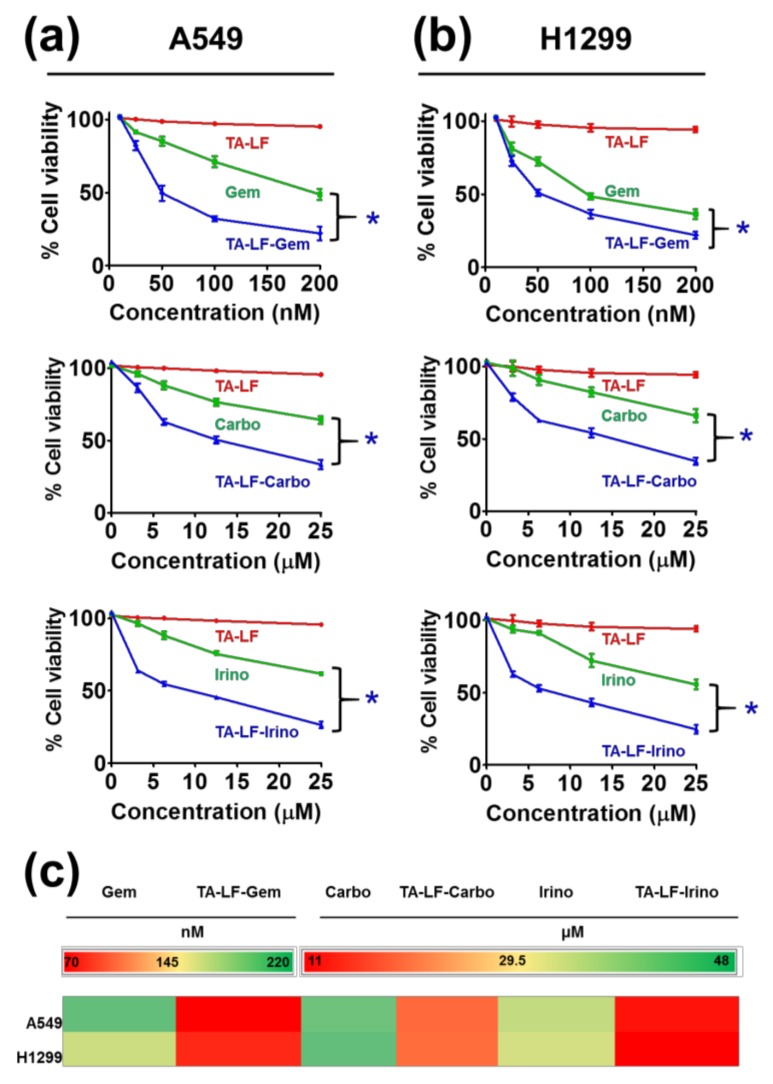
TA-LF complexes promotes delivery of encapsulated therapeutic drugs to LC cells. MTS assay of drug-encapsulated TA-LF complexes against (**a**) A540 and (**b**) H1299 LC cells. Cells (5 × 103) were seeded in a 96-well plate and left overnight for cell attachment to the plate, the cells were treated with indicated concentrations of gemcitabine, carboplatin, and irinotecan and their respective drug-encapsulated TA-LF complexes for 48 h. Cell viability was determined using MTS assay. TA at the concentrations used to make TA-LF complexes were used as controls. The data were presented in the form of line graphs as percent viable cells compared to untreated cells in medium. Data presented as mean ± SEM (each treatment, *n* = 6). Cytotoxicity of TA-LF-drug formulations were significant compared to free drugs (* *p*  <  0.05). (**c**) IC_50_ values of drug vs. drug-encapsulated TA-LF assemblies presented as heat map. IC_50_ values were calculated using GraphPad software (La Jolla, CA, USA).

To study the potential enhancement of anti-cancer activity when encapsulated in TA-LF assemblies, we employed an MTS assay against two LC cell lines. This assay measures viability of the cancer cells based on mitochondrial respiratory activity. There is no effect on A549 and H1299 cells when treated with free TA-LF complexes (without drug) (Figure 5a,b, red color lines). A clear dose-dependent effect was observed with free pharmaceutical drugs (gemcitabine, carboplatin, and irinotecan) against cancer cells (Figure 5a,b, green color lines). TA-LF complexes showed more significant growth-inhibiting effects than free drugs (Figure 5a,b, blue color lines). This indicates that TA-LF assemblies promote interaction and delivery of drug(s) to the cells tested. In all drug-encapsulated TA-LF assemblies, there was at least 2–2.5 fold reduction in their IC_50_ values compared to free drugs (Figure 5c). 

**Figure 6:**
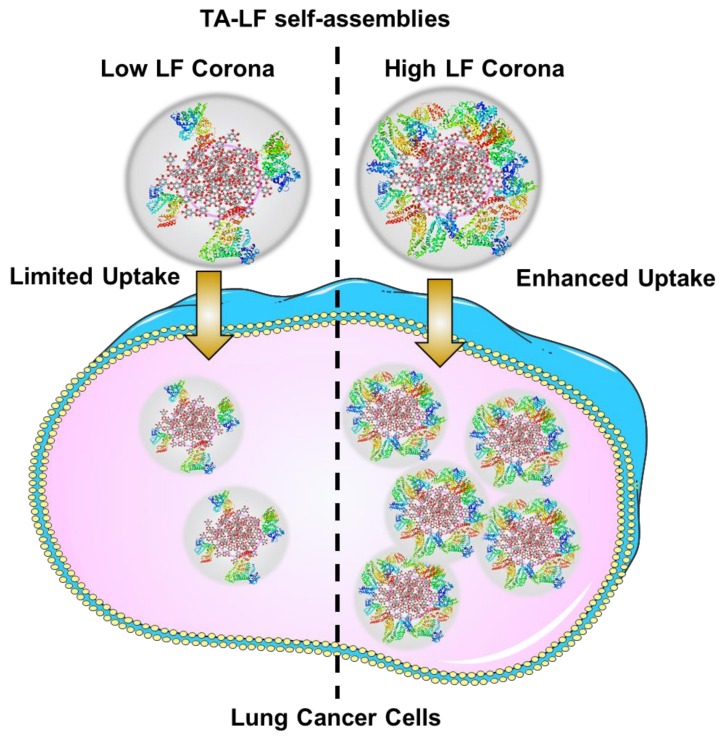
Schematic representation of LF corona influencing delivery of TA-LF assemblies to LC cells.

An effective chemotherapy not only lowers systemic toxicity, but also lowers the therapeutic dose. This study supports the hypothesis that TA-LF can be implemented as an efficient drug carrier (Figure 6). The developed method may overcome conventional chemotherapy and its associated drawbacks such as modest benefit and drug resistance. Lately, nanomedicine (for example, Abraxane^TM^, a paclitaxel self-assembled human serum albumin formulation) represents a universal drug delivery platform for the treatment of LC, which makes chemotherapeutic drugs more efficient in treatment regimens. Likewise, our TA-based drug formulations might serve as a better therapeutic modality for LC. Our future investigation on these formulations will include studies regarding superior molecular mechanisms and pre-clinical animal studies. 

## 4. Conclusions

Overall, this investigation revealed a naturally occurring water-soluble polyphenolic drug carrier and its interaction with LF proteins. The study demonstrated that TA exhibits superior and well-defined protein corona formation. This was demonstrated by particle size, zeta potential, morphology, cellular internalization, and anti-cancer activity assays. Further, the results can be extrapolated to physiological relevance of the adsorption of LF and reduction of surface tension. Therefore, TA may be proposed as a promising carrier for future research into drug delivery applications.

## Supplementary Materials

The following are available online at http://www.mdpi.com/1999-4923/10/3/111/s1, Figure S1: FT-IR spectrum of LF, Figure S2: Supernatant and pellet TA-LF analysis on nitrocellulose membrane, Figure S3: Particle size and zeta potential of TA and LF.
