# Supplementary material for: Tannic Acid-Lung Fluid Assemblies Promote Interaction and Delivery of Drugs to Lung Cancer Cells
Source: Pharmaceutics. 2018 Aug 1;10(3):111. doi: 10.3390/pharmaceutics10030111 (PMC6161105; doi:10.3390/pharmaceutics10030111)
Supplement: Supplementary file 1 [file pharmaceutics-10-00111-s001.pdf]

Supplementary Materials: Tannic Acid-Lung Fluid Assemblies Promote Interaction and Delivery of Drugs to Lung Cancer Cells

Elham Hatami [1], Prashanth K.B. Nagesh [1], Pallabita Chowdhury [1], Subhash C. Chauhan [1], Meena Jaggi [1], Amali E. Samarasinghe [2] and Murali M. Yallapu [1,*]

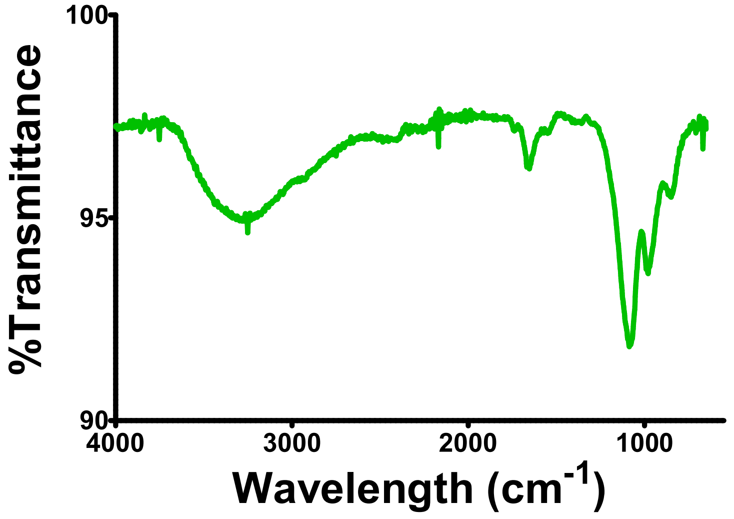

Figure S1. FT-IR of lung fluid. Protein characteristics peaks of the amide I band at 1655 cm^{-1} (mainly C–O stretch) and amide II band at 1525 cm^{-1} (C–N stretching and N–H bend) in LF. The other peaks at 3290 and 1075 cm^{-1} corroborates to N–H and C–N stretch of the aliphatic amine.

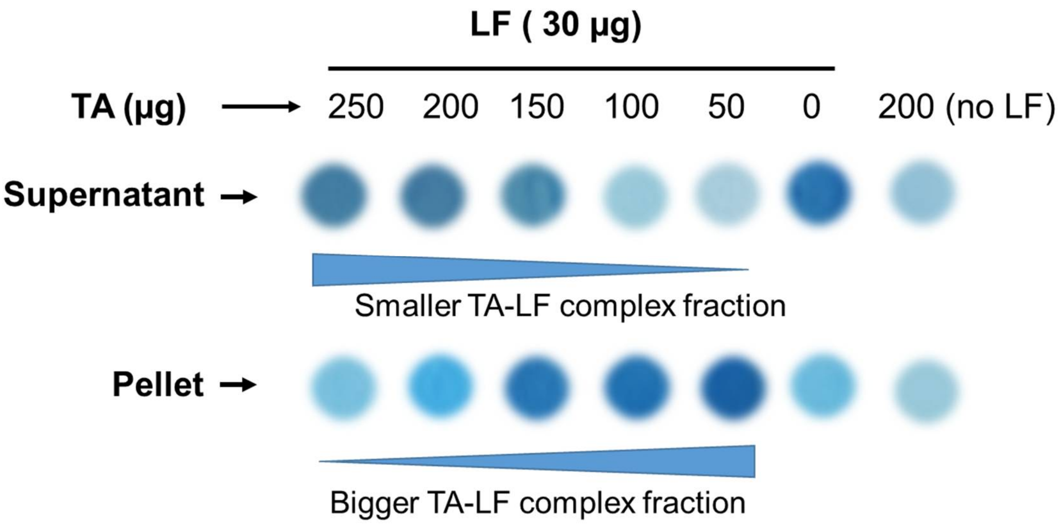

Figure S2. TA-LF complexes were separated by centrifugation at 5,000 rpm and supernatant and pellet TA-LF complexes were used for protein density probing on nitrocellulose membrane as mentioned in section 2.5 in the manuscript.

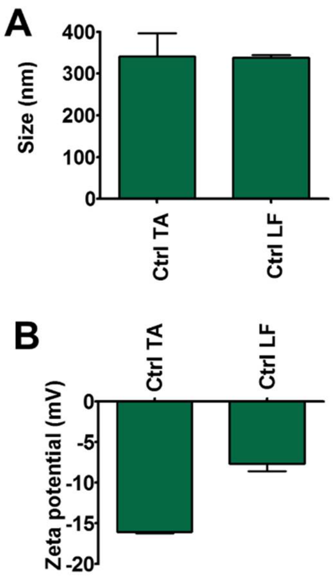

Figure S3. DLS data for (**A**) Particle size and (**B**) zeta potential of TA and LF. Measurements were performed using Zetasizer.
